# Determinants of self-rated health among shanghai elders: a cross-sectional study

**DOI:** 10.1186/s12889-017-4718-5

**Published:** 2017-10-13

**Authors:** Weizhen Dong, Jin Wan, Yanjun Xu, Chun Chen, Ge Bai, Lyuying Fang, Anjiang Sun, Yinghua Yang, Ying Wang

**Affiliations:** 1 Department of Sociology and Legal Studies, University of Waterloo, 200 University Avenue West, Waterloo, ON N2L 3G1 Canada; 2 Research Institute of Health Development Strategies, Fudan University, 130 Dong An Road, Shanghai, 200032 China; 3 School of Humanities and Management, Wenzhou Medical College, Wenzhou, Zhejiang 325035 China; 4 Wenzhou Medical University Chashan Campus, Chashan University Town, Tongren Building 7B302, Wenzhou, Zhejiang 325035 China; 5 Shanghai Municipal Center for Disease Control & Prevention, yangyinghua@scdc.sh.cn, No. 1380 Zhongshan West Road, Shanghai, 200336 China

**Keywords:** Self-rated Health, Elders, Health Status, Shanghai

## Abstract

**Background:**

As the most populous nation in the world, China has now becoming an emerging ageing society. Shanghai is the first city facing the challenge of ageing demographics. Against this background, a study that employs self-rated health (SRH) assessment system was designed to explore the health status of Shanghai elders, and learn their attitudes toward health issues; as well as to investigate the determinants of SRH among Shanghai elders. Understanding SRH is crucial for finding appropriate solutions that could effectively tackle the increasing eldercare demand.

**Methods:**

This study adopted a quantitative research strategy. Using a multistage stratified cluster sampling method, we conducted a questionnaire survey in August 2011 in Shanghai, which collected 2001 valid survey responses. SRH assessments were categorized by five levels: very good, fairly good, average, fairly poor, or poor. The respondents’ functional status was evaluated using the Barthel index of activities for daily living. In the data analysis, we used chi-squared test to determine differences in socio-demographic characteristics among various groups. Along with statistics, several logistic regression models were designed to determine the associations between internal influence factors and SRH.

**Results:**

Younger age (χ^2^ = 27.5, *p* < 0.05), male sex (χ^2^ = 11.5, *p* < 0.1), and living in the suburbs (χ^2^ = 55.1, *p* < 0.05) were associated with better SRH scores. Higher SRH scores were also linked with health behaviour of the respondents; namely, do not smoke (χ^2^ = 18.0, p < 0.1), do not drink (χ^2^ = 18.6, p < 0.1), or engage in regular outdoor activities (χ^2^ = 69.3, p < 0.05). The respondents with better social support report higher SRH scores than those without. Respondents’ ability to hear (χ^2^ = 38.7, p < 0.05), speak (χ^2^ = 16.1, p < 0.05) and see (χ^2^ = 78.3, p < 0.05) impacted their SRH scores as well. Meanwhile, chronic illness except asthma was a major influence factor in low SRH score. Applying multiple regression models, a series of determinants were analysed to establish the extent to which they contribute to SRH. The impact of these variables on SRH scores were 6.6% from socio-demographic and health risk behaviours, 2.4% from social support, 8.5% from mental health, 20% from physical conditions, and13% from chronic diseases.

**Conclusions:**

This is the first study that examines the determinants of SRH among Shanghai elders. Nearly 40% of our study’s respondents reported their health status as “good”. The main determinants of SRH among elders include living condition, health risk behaviour, social support, health status, and the economic status of the neighbourhood.

## Background

Population ageing has been an ongoing trend globally in recent years. By the year 2015, there were more than 826 million people over 60 years of age in the world—a number projected to reach nearly 1.4 billion by 2030 [1–3]. The pace of population ageing is much rapid in China than many other high-income or low-and middle-income countries [4]. According to China’s sixth National Population Census, 13.3% of the nation’s entire population was aged ≥60 years in 2010 [5], and among these, 12.3% were aged ≥80 years [6]. In the next 25 years, the percentage of people in China aged 60 years or over is expected to be more than double, reach 28% (402 million) in 2040. In contrast, for the proportion of the population aged over 60 years to double from 7% to 14% took France, Sweden and the US 115 years, 85 years and 69 years respectively [4].

Shanghai is known for its dynamic economy and prolific resources. In 2016, its GDP was ¥2746.6 billion, an increment of ¥250.1 billion of the previous year, ranked No.1 among all cities of China. The annual GDP increase in Shanghai was 10.02%, while Beijing was 8.41% [7]. Shanghai was also the first city to enter ageing society in China. The average life expectancy in Shanghai reached 82.75 years of age by the end of 2015 (male 80.47, female 85.09). In the same year, 4.36 million (30.2%) of the city’s registered residents were aged ≥60 years, the highest proportion of older residents among China’s regional jurisdictions. Among them, 780, 500 were aged ≥80 years [8]. It is estimated that the proportion of elders among Shanghai residents could exceed 34% by the year 2020 [9], and 44.5% by the year 2050, which is higher than the estimated proportion of elders in Japan [10].

Population aging brings about major challenges to health and social care. Learn the health status of the elderly is vital for informing the development of disease prevention and health promotion programs, and healthcare and social services delivery that can enhance their life satisfaction and quality of life [11]. Self-rated health (SRH) is a useful assessment of health derived from asking individuals to rate their own general health on a five-point scale. SRH is considered by the World Health Organization to be an important indicator of population health and healthy life expectancy [12]. Numerous studies show that the age, gender, income, education [13], life style, psychological well-being [14], social support such as children’s accompany and emotional support [15], chronic diseases, physical and instrumental functioning [16, 17] are associated with self rate health [18].

According to health screening data, only about 20%–25% of elderly people have no vital diseases [19]. After over three decades of “one-child” policy, a period in which many young adults have sought opportunities far from their hometowns, the traditional intergenerational relationship between the elderly and their children has changed, as has the traditional family structure [20]. Thus, it is important to find ways in which society can meet the increasing needs of the elders. Employing SRH measures to investigate Shanghai elders’ view on their own health status will help to explore what are the determinants of SRH among Shanghai elders as well.

### Objectives

1) to assess the SRH of community-dwelling elders in Shanghai; 2) to examine the effects on individual older persons’ SRH from demographic characteristics, health risk behaviours, physical/mental health condition, social support, and neighbourhood socioeconomic status, etc.; 3) to determine the main determinants affecting Shanghai elders’ SRH; and 4) to explore the subsequent public health and policy implications. This study will also extend the literature on SRH, particularly with regard to the ageing population in Shanghai.

## Methods

### Sampling

This study conducted a questionnaire survey that employed a multistage stratified cluster random sampling method. The sample was selected from Shanghai residents who were ≥60 years of age. In China, people who have reached 60 years of age are classified as elders [4]. Based on hypothesis testing, and considering the types I (0.05) and II (0.1) statistical error, the appropriate sample size for this project was considered to be 1756. This study obtained 2001 valid survey responses.

The multistage stratified cluster random sampling process included the following:

First, all of Shanghai’s 18 districts were divided into three groups according to their economic status – high, medium and low. Then, two districts each were randomly selected from the three groups, namely: Pudong and Changning (high), Hongkou and Putuo (medium), and Jinshan and Chongming (low). Based on the sample districts’ population size, 832, 199, 291, 274, 157, and 248 elders should be selected from the six districts respectively. Second, all neighbourhood communities in the six districts were ranked based on economic status (high, medium, or low), and one medium-level community was selected from each. Third, the selected communities provided the lists of their elder residents’ names, and participants were selected randomly from the lists based on the required sample size of the study.

### Data collection and ethics considerations

This study’s ethics review application was conducted and approved by the Research Ethics Board of Fudan University. The project team conducted questionnaire survey from June to August of 2011. All participants gave written informed consent before the survey took place. The investigators filled out the questionnaires during the survey on behalf of the respondents. The respondents were given the option of leaving the study at any time with no question asked; but none did.

### Measurement

#### Dependent variable

SRH among Shanghai elders is the dependent variable in this study. SRH was assessed by asking the question: “How do you rate your health: very good, fairly good, average, fairly poor, or poor?” For cross-tabulations, SRH was categorized as good (very good and fairly good), average, and poor (fairly poor and poor); and for logistic regression analyses, it was categorized as good (very good and fairly good), and not good (average, fairly poor and poor).

#### Independent variables

There are 57 aspects in five sections in the questionnaire, and details are as follows:

**Characteristics:** residence location (urban, suburbs or outer suburbs); gender (male or female); age group (60–64, 65–69, 70–74, 75–79, and ≥80 years), and educational attainment (illiterate, primary, middle/high school, college or above).

**Health risk behaviour:** cigarette smoking (never, occasionally, often, has quit); alcohol drinking (never, occasionally, often, has quit); outdoor activities (yes, no).

**Social support:** marital status (widowed, married, single/divorced); source of income (pension, salary/savings, family allowance, other); living situation (non-empty nester, empty nester); meeting children (daily, weekly, monthly, yearly, <1 time/year); meeting neighbours (weekly, monthly, yearly, never); meeting relatives/friends (weekly, monthly, yearly, never); community activities (weekly, monthly, yearly, never).

**Mental health status:** feelings of loneliness (never, sometimes, always); nervousness/anxiety (never, sometimes, always); satisfaction with life (good, fair, poor).

**Physical health status:** changes in physical health status (better, unchanged, worse, variable); hearing (deaf, some hearing, able); ability to speak (yes, no); vision (blind, some vision, sighted). Furthermore, the Barthel index of activities of daily living (ADLs) was adopted for assessing the functional status of the respondents in the study. ADLs scores were classified as complete, partial, or almost no ability to perform activities.

The index consists of six measures: bathing and showering, dressing, self-feeding, functional mobility, personal hygiene and grooming, and toilet hygiene. The respondents were asked their degree of dependency in eating, personal hygiene, bathing/showering, dressing/undressing, walking, use of stairs, and toilet hygiene [21].

**Chronic disease:** Chronic diseases in the study were those diagnosed by physicians or symptoms described by the respondents, including hypertension, heart disease, diabetes, cataract, cerebrovascular disease, bronchitis, gastroenteritis, intervertebral disc disease, cardiovascular disease, and asthma. The survey responses were as follows: chronic disease (yes, no); hypertension (yes, no); heart disease (yes, no); diabetes (yes, no); cataracts (yes, no); cerebrovascular disease (yes, no); bronchitis (yes, no); gastroenteritis (yes, no); intervertebral disc disease (yes, no) cardiovascular disease (yes, no); asthma (yes, no); total number of chronic diseases (0, 1, 2, or ≥3).

### Statistical analysis

The SPSS statistical package (version 16.0 for Windows) was used to analyse the survey data. Mean S.D. was used for descriptive statistics. The chi-squared test was used to determine the differences in socio-demographic characteristics among different groups in the data analysis. Significance was defined as *p* < 0.05. The relationship between SRH score and respondents’ socio-demographic characteristics, health risk behaviours, social support, mental health status and physical health status were tested using the chi-squared test. A series of logistic regression models were used to determine the independent association between SRH and its influencing factors.

## Results

This study included 2001 respondents from different residential communities of Shanghai (Table 1). The mean age was 71.6 years. 324 of them or 16.2% of the total in the sample were aged ≥80 years. The gender ratio was 2:3 (841 male to 1153 female). The ratio of dwelling locations was 2:2:1 for downtown, suburbs and outer suburbs respectively. 39.4% of them received high school or higher education, and 28.7% were illiterate (see Table 2).

**Table 1:** Selected communities and characteristics of respondents

District	Pudong	Changning	Hongkou	Putuo	Jinshan	Chongming
Male	339	83	109	129	76	107
Female	493	115	165	163	78	140
Age ≥ 60	715	165	211	244	139	200
Age 80~	117	32	63	48	18	47

**Table 2:** Socio-demographic characteristics and health risk behaviours of respondents

SRH		Good		Fair		Poor		Total	
		*N*	%	*N*	%	*N*	%	*N*	%
socio-demographic									
residence^**^	urban	272	35.6	390	51.1	101	13.2	763	38.2
	suburbs	428	51.4	320	38.5	84	10.1	832	41.7
	outer suburbs	188	46.8	144	35.8	70	17.4	402	20.1
gender^*^	male	409	48.6	342	40.7	90	10.7	841	42.2
	female	479	41.5	511	44.3	163	14.1	1153	57.8
age^**^	60~	320	49.8	259	40.3	64	10.0	643	32.2
	65~	161	47.5	140	41.3	38	11.2	339	17.0
	70~	139	44.3	134	42.7	41	13.1	314	15.7
	75~	152	40.4	172	45.7	52	13.8	376	18.8
	80~	116	35.8	148	45.7	60	18.5	324	16.2
education level	illiterate	243	42.6	241	42.2	87	15.2	571	28.7
	primary	280	44.1	266	41.9	89	14.0	635	31.9
	Middle/high	314	46.7	295	43.9	63	9.4	672	33.7
	College or above	50	43.9	49	43.0	15	13.2	114	5.7
health risk behaviours									
smoking^*^	never	677	43.5	676	43.4	203	13.0	1556	77.9
	occasionally	45	53.6	32	38.1	7	8.3	84	4.2
	often	131	52.6	93	37.3	25	10.0	249	12.5
	quit	35	32.4	53	49.1	20	18.5	108	5.4
drinking^*^	never	669	42.7	680	43.5	216	13.8	1565	78.4
	occasionally	77	51.3	65	43.3	8	5.3	150	7.5
	often	32	59.3	20	37.0	2	3.7	54	2.7
	quit	110	48.2	89	39.0	29	12.7	228	11.4
outdoor activities^**^	yes	603	51.8	453	38.9	108	9.3	1164	58.4
	no	284	34.2	401	48.3	145	17.5	830	41.6

^**^means *p* < 0.05

^*^means *p* < 0.1

### Health risk behaviours have a positive association on SRH

Most of the respondents were non-smokers (77.9%) and non-drinkers (78.4%); and more than a half (58.4%) took part in regular outdoor physical exercise. There was a linear relationship between age and SRH. Table 2 shows that younger age (χ^2^ = 27.5, *p* < 0.05), male sex (χ^2^ = 11.5, *p* < 0.1), and living in suburban areas (χ^2^ = 55.1, p < 0.05) were associated with better SRH scores.

Another group with better SRH scores was those who smoke cigarettes (χ^2^ = 18.0, p < 0.1) or drink alcohol (χ^2^ = 18.6, p < 0.1). These scores were better regardless of whether they smoked/drank occasionally or regularly, or frequency of participation in outdoor activities (χ^2^ = 69.3, p < 0.05). Only 10% of regular cigarette smokers, 3.7% of regular alcohol drinkers, and 9.3% of physically active respondents rated their health status as “poor”.

### The stronger the social support, the higher the SRH score

The association between social support and mental condition and SRH scores was cross-tabulated and tested using the chi-squared test. The results show that the majority (75.9%) of the respondents were married, and 21.6% were widowed. The main source of income for 86.1% of respondents was a government pension. More than three-fifths of them (65.3%) were empty nesters. A similar proportion, 65.2%, reported meeting with their children daily and nine out of ten interacted with their neighbours weekly, and 22.4% met with their relatives and friends on a weekly basis. In terms of mental health, 83% of the respondents reported that they never felt lonely and 89.2% of them had never felt nervous or anxious. Only 5.1% reported that they were not very satisfied with their lives.

As shown in Table 3, respondents with better social support tended to have better SRH scores. For example, those who received a pension (χ^2^ = 28.8, *p* < 0.05) and met more frequently with their children (χ^2^ = 21.1, *p* < 0.1), neighbours (χ^2^ = 25.3, p < 0.05), and relatives and friends (χ^2^ = 41.6, p < 0.05) tended to have significantly better SRH scores.

**Table 3:** Social support and mental health status of respondents

SRH		Good		Fair		Poor		total	
		*N*	%	*N*	%	*N*	%	*N*	%
social supports									
marital status	widowed	172	40.1	191	44.5	66	15.4	429	21.6
	married	691	45.8	637	42.2	182	12.1	1510	75.9
	single/divorced	22	43.1	23	45.1	6	11.8	51	2.6
source of income^**^	pension	782	46.2	711	42.0	198	11.7	1691	86.1
	salary/savings	46	31.9	83	57.6	15	10.4	144	7.3
	family	20	35.7	23	41.1	13	23.2	56	2.8
	other	29	39.2	28	37.8	17	23.0	74	3.8
living situation	non-empty nester	308	44.5	292	42.2	92	13.3	692	34.7
	empty nester	579	44.4	562	43.1	163	12.5	1304	65.3
meeting children^*^	daily	600	46.8	538	42.0	143	11.2	1281	65.2
	weekly	170	41.6	179	43.8	60	14.7	409	20.8
	monthly	74	38.1	92	47.4	28	14.4	194	9.9
	yearly	29	43.9	21	31.8	16	24.2	66	3.4
	<1 time/year	4	25.0	10	62.5	2	12.5	16	0.8
meeting neighbours^*^	weekly	821	45.8	762	42.5	210	11.7	1793	89.8
	monthly	24	31.6	39	51.3	13	17.1	76	3.8
	yearly	7	43.8	6	37.5	3	18.8	16	0.8
	never	36	32.4	47	42.3	28	25.2	111	5.6
meeting relatives/friends^**^	weekly	249	55.6	162	36.2	37	8.3	448	22.4
	monthly	143	46.0	137	44.1	31	10.0	311	15.6
	yearly	363	41.7	383	44.0	124	14.3	870	43.6
	never	133	36.2	172	46.9	62	16.9	367	18.4
community meeting^**^	weekly	151	50.3	104	34.7	45	15.0	300	15.1
	monthly	81	52.3	62	40.0	12	7.7	155	7.8
	yearly	110	52.9	86	41.3	12	5.8	208	10.5
	never	544	41.0	598	45.1	185	13.9	1327	66.7
mental status									
loneliness^**^	never	798	48.2	684	41.3	175	10.6	1657	83.0
	sometimes	67	25.1	143	53.6	57	21.3	267	13.4
	always	23	31.9	26	36.1	23	31.9	72	3.6
nervousness/ anxiety^**^	never	841	47.2	749	42.1	190	10.7	1780	89.2
	sometimes	40	22.9	92	52.6	43	24.6	175	8.8
	always	6	15.0	12	30.0	22	55.0	40	2.0
satisfied with life^**^	good	632	58.0	367	33.7	91	8.3	1090	54.7
	fair	239	29.8	455	56.7	108	13.5	802	40.2
	poor	16	15.7	30	29.4	56	54.9	102	5.1

^**^means *p* < 0.05

^*^means *p* < 0.1

### Better physical health is positively associated with better SRH scores

Most (98.1%) of the respondents in this study were able to live independently. Over the past year, 64% reported had no change in physical condition and less than 29.2% of them felt some decline of health in the past year. One fifth noticed a decline in their hearing ability and 5% were unable to hear. 6.5% of the respondents had difficulty in speaking and 28.5% of them had vision issues (see Table 4).

**Table 4:** Physical health condition of the respondents

SRH		Good		Fair		Poor		Total	
		*N*	%	*N*	%	*N*	%	*N*	%
Physical health									
ADL	Complete	887	45.3	844	43.1	229	11.7	1960	98.1
	Partial	1	3.0	10	30.3	22	66.7	33	1.7
	No ability	0	0.0	0	0.0	4	100.0	4	0.2
physical health changes**	Better	49	49.5	39	39.4	11	11.1	99	5.0
	Unchanged	686	53.8	529	41.5	61	4.8	1276	64.0
	Worse	146	25.1	259	44.6	176	30.3	581	29.2
	variable	7	18.9	25	67.6	5	13.5	37	1.9
hearing**	deaf	29	29.0	51	51.0	20	20.0	100	5.0
	some	140	36.6	170	44.4	73	19.1	383	19.2
	able	719	47.6	632	41.9	158	10.5	1509	75.8
speaking**	yes	52	40.3	46	35.7	31	24.0	129	6.5
	no	834	44.8	807	43.3	222	11.9	1863	93.5
vision**	sighted	701	49.4	588	41.4	131	9.2	1420	71.5
	some vision	143	32.1	207	46.5	95	21.3	445	22.4
	blind	38	31.4	56	46.3	27	22.3	121	6.1
Chronic disease									
with chronic disease**	Yes	587	38.1	711	46.1	243	15.8	1541	77.2
hypertension**	Yes	402	39.4	468	45.9	150	14.7	1020	51.1
heart disease**	Yes	117	26.9	204	46.9	114	26.2	435	21.8
diabetes**	Yes	89	30.7	141	48.6	60	20.7	290	14.5
cataract**	Yes	50	28.9	93	53.8	30	17.3	173	8.7
cerebrovascular disease**	Yes	33	22.1	65	43.6	51	34.2	149	7.5
bronchitis**	yes	34	28.3	63	52.5	23	19.2	120	6.0
gastroenteritis**	yes	29	25.7	55	48.7	29	25.7	113	5.7
intervertebral disc disease**	yes	36	32.1	46	41.1	30	26.8	112	5.6
cardiovascular disease*	yes	23	40.4	26	45.6	8	14.0	57	2.9
asthma	yes	19	36.5	19	36.5	14	26.9	52	2.6
number of chronic diseases/person	0	301	66.0	143	31.4	12	2.6	456	22.8
	1	336	49.9	284	42.1	54	8.0	674	33.8
	2	151	34.8	221	50.9	62	14.3	434	21.7
	3 or more	100	23.1	206	47.6	127	29.3	433	21.7

^**^means *p* < 0.05

^*^means *p* < 0.1

The overall prevalence of chronic diseases among the respondents was quite high (77.2%), however. The 10 most commonly reported chronic diseases were: hypertension (51.1%), heart disease (21.8%), diabetes (14.5%), cataracts (8.7%), cerebrovascular disease (7.5%), bronchitis (6.0%), gastroenteritis (5.7%), intervertebral disc disease (5.6%), cardiovascular disease (2.9%), and asthma (2.6%). The percentage of respondents reporting having three or more of the diseases was 22.7%, and a similar percentage have two diseases.

Chi-square analysis found that the respondents’ ability in hearing (χ^2^ = 38.7, *p* < 0.05), speaking (χ^2^ = 16.1, p < 0.05) or vision (χ^2^ = 78.3, p < 0.05) affects their SRH negatively. Chronic illnesses (except asthma) also impact SRH significantly; and the degree of influence on SRH was associated with the number of reported diseases: more diseases also resulted in a poorer SRH score.

#### Models’ testing on the determinants of SRH

Table 5 reports a series of logistic regression models of predicted odds ratios of SRH.

**Table 5:** Multiple Logistic Regression of Factors Influencing SRH of Respondents

Variables	Model 1	Model 2	Model 3	Model 4	Model 5	Model 6
Socio-demographics						
Urban (outer suburb)	1.664**	1.933**	1.554**	1.532**	1.665**	1.689**
Suburb (outer suburb)	0.808	1.025	0.813	0.789	0.767**	0.984
Female (male)	1.18	1.166	1.117	1.221	1.11	1.129
65–69 years old (60–64)	1.097	1.11	1.129	1.086	0.972	1.06
70–74 years old (60–64)	1.136	1.134	1.16005	1.055	0.943	0.968
75–79 years old (60–64)	1.151	1.163	1.137	1.086	0.954	0.996
≥ 80 years old (60–64)	1.291	1.329	1.268	1.106	1.058	1.035
1–6 years of schooling (0)	1.052	1.091	1.098	0.993	0.989	1.059
7–12 years of schooling (0)	0.83	0.84	0.812	0.756	0.815	0.771
13- years of schooling (0)	0.744	0.678	0.785	0.642	0.721	0.671
Health risk behaviours						
smoke sometimes (never)	0.931	1.015	0.880	0.993	0.932	1.056
always smoke (never)	1.04	1.036	0.925	1.087	1.119	1.049
quit smoking (never)	2.329**	2.396**	2.139**	2.204**	2.321**	2.212**
drink sometimes (never)	0.776	0.751	0.712	0.861	0.819	0.791
always drink (never)	0.645	0.608	0.638	0.586	0.729	0.618
quit drinking (never)	0.706	0.675**	0.729	0.736	0.665**	0.686
outdoor activities (no)	1.923**	1.755**	1.604	1.809	1.794**	1.412**
Social support						
in marriage (widowed)		1.172				1.279
unmarried (widowed)		1.108				1.221
empty nesters (Non)		1.063				1.002
work pay/savings (pension)		2.172**				1.601**
from family (pension)		1.401				1.225
others (pension)		1.539				1.702
children/week (/day)		1.102				0.991
children/month (/day)		1.279				1.155
children/year (/day)		0.954				0.808
children < 1 / year (/day)		2.71				1.198
neighbour/month (/week)		1.417				1.7
neighbour/year (/week)		0.58				0.676
neighbour/none (/week)		1.159				1.089
relative or friend /month (/week)		1.404**				1.658**
relative or friend /year (/week)		1.626**				1.845**
relative or friend/none (/week)		1.438**				1.627**
community/month (/week)		0.848				0.778
community/year (/week)		0.812				0.811
community/none (/week)		1.195				1.056
Mental condition						
loneliness (no)			3.213**			1.310
nervous/anxiety (no)			6.227**			1.906**
general feeling (good)			0.996			3.059**
bad feeling (good)			1.967**			4.415**
Physical condition						
Tool use and ADL						
use auxiliary tools (no)				1.343		1.114
ADL (no)				16.062**		11.14**
Chronic diseases						
hypertension (no)					0.778**	0.751**
heart disease (no)					0.517**	0.553**
Diabetes (no)					0.623**	0.572**
Cataract (no)					0.592**	0.673
Cerebrovascular disease (no)					0.438**	0.513**
Bronchitis (no)					0.667	0.608
Gastroenteritis (no)					0.488**	0.565**
intervertebral disc disease (no)					0.708	0.787
cardiovascular disease (no)					0.877	0.821
Asthma (no)					0.428	0.39
Constant	−0.268	−1.141	−0.853	−0.354	−1.247	2.876
χ^2^	134.711	179.683	322.045	137.527	395.501	418.466
df	19	38	23	21	41	54
Sig.	*P* < 0.05	*P* < 0.05	*P* < 0.05	*P* < 0.05	*P* < 0.05	*P* < 0.05
R^2^	0.066	0.09	0.151	0.07	0.196	0.206

Model 1 shows that the location of *residence* is a significant determinant of the elders’ SRH. Respondents who are living in outer suburbs are more likely to have better SRH score. Health risk behaviours such as smoking, drinking and outdoor exercise were significantly and positively associated with their SRH. Those who had quit smoking were 2.3 times as likely to have poor SRH than the ones who had never smoked.

Model 2 indicates that respondents who enjoy better social support are more likely to have higher SRH score. Meeting with family, relatives and friends more often and taking part in more community activities was associated with higher SRH score. Those who receiving a pension are 2.2 times as likely to have higher SRH score than those living on savings or allowances from their children. However, marital status and living arrangements have no significant association with SRH.

Model 3 shows that the elders who are satisfied with their lives are twice as likely to have higher SRH scores. Meanwhile, feelings of loneliness are 3.2 times as likely to have low SRH, and those who reported feeling nervous or anxious are 6.2 times as likely to have low SRH score.

Model 4 shows that ADLs disability and using assistive tools in daily lives impact SRH score. The respondents who have physical functional challenges and required assistance in their daily lives are 16.1 times as likely to have low SRH score than those who live independently and without physical disability.

Model 5 includes the variables of 10 chronic diseases. It shows that these diseases are all significantly associated with SRH. Those with one or more chronic diseases are more likely to have lower SRH score.

Model 6 includes all of the variables in the survey. Overall, the respondents with better SRH scores are 1.7 times as likely to be living in the outer suburbs, 2.2 times as likely to be non-smokers, 1.4 times as likely to have had regular outdoor exercises, and those who meet with family, relatives or friends more often. While respondents with lower SRH scores are 1.9 times as likely to feel nervous or anxious, 4.4 times as likely to be dissatisfied with their lives, and 11.1 times as likely to be ADLs disabled and/or have been diagnosed with chronic illnesses such as hypertension, cardiovascular disease, diabetes, cerebrovascular disease and gastroenteritis.

The results of multiple logistic regressions show that socio-demographic and health risk behaviours only made up 6.6% of the total contribution to SRH. When combined with mental health variables (*Model 3*), it increased to 15.1%. Adding chronic diseases (*Model 5*), it became 19.6%. And all socio-demographic and health risk variables (*Model 6*) contributed 20.6% to SRH.

The models above show that among various determinants of SRH of the Shanghai elders, 6.6% is from socio-demographic and health risk behaviours, 2.4% is from social support, 8.5% is from mental health, 20.0% is from physical conditions, and 13.0% from chronic diseases.

## Discussion

SRH was initially used by Mossey and Shapiro for predicting 7-year survival odds; and it became widely used as an indicator of population health afterwards [22]. According to Jylha, SRH is an individual and subjective conception that constitutes a crossroad between the social world and psychological experience on one hand, and the biological world on the other [23]. Scholars in different nations investigated the correlates of SRH based on Jylha’s theory; and French and Browning concluded that correlates of SRH are similar across continents [24]. This study examined the determinants of SRH among elders in Shanghai, which provides new evidence on whether the correlates of SRH similarities are greater than the differences between regions.

Multiple cross-sectional studies have been done previously on associations between SRH and health status as well as other factors. Most of these studies found that socio-demographic characteristics such as age, gender, education level, location of residency are related to SRH [25, 26]. Lower educational attainment is associated with lower SRH score [27], physical inactivity is associated with a decline in SRH score [28], and smoking is associated with lower SRH score. Modest alcohol intake and more social support are associated with higher SRH score [29–31]; while deterioration in physical [32, 33] or mental [32] health would significantly lower one’s SRH score.

In our examination of the relationships between SRH and socio-demographic factors, health risk behaviours, physical and mental health condition, and social support, nearly 40% of the respondents reported good SRH. This ratio is very close to the findings from some developed countries (i.e. 39.0% in Finland [34] and 39.6% in Spain [35]), and is considerably higher than similar studies’ findings from other parts of China (36.4%; 35.3%) [36, 37]. Shanghai’s relatively better socio-economic conditions and healthcare access may have contributed to the elders’ better SRH outcome [38].

Examining the extent of various determinants and their association with SRH can help to assess their relative importance in people’s lives, and develop more effective strategies to improve population health. Consistent with prior studies’ findings, multiple logistic regression analysis in this study indicated that physical health matters more than socio-demographic and risk health behaviours, which means that the SRH scores of the respondents, to a great degree, was affected by their physical health, which reflect their true health conditions. [39, 40].

Both single factor analysis and multiple regression analysis show that those respondents who lived in outer suburbs have higher SRH scores, while those who live in downtown have the poorest SRH. Meanwhile, the elders who had quit smoking, but not those who are still smoking, are more likely to have lower SRH scores. It may be assumed that those who quit smoking was due to declining of physical health. These new findings, as well as those of previous studies, indicate that health risk behaviour such as drinking and smoking have positive effects on SRH. Respondents who participated in outdoor activities regularly are also more likely to have higher SRH scores, which means that increasing the elders’ physical activities participation improves their SRH [41].

Further analysis found that social support, health risk behaviours, health status and neighbourhood economic status all impact on Shanghai elders’ SRH.

### Social support

Respondents’ social support situation has a direct impact on their SRH. Generally, those with stronger social support networks tend to have better SRH scores. This demonstrates that being involved in more social function (such as interpersonal communication and emotional attachment with others) results in a better SRH score. It also suggests that SRH may be closely tied with mental health and overall wellbeing. These findings are also quite consistent with some previous studies [42, 43].

However, financial support from family members did not seem to have positive association with SRH. This finding is very different from previous studies’ findings, which found that financial support from family members tended to have a positive association with SRH [44]. While this new finding deserving further investigation, some assumptions could be made as spring board for such new studies: 1) they did not need financial support, 2) they did not like to be a burden of their respective families, or 3) they value emotional support more than financial assistance. Shanghai is a relatively affluent city and its residents are relatively well-off. According to 2016 Pension policy launched by Social Security Bureau in Shanghai, people over 60 years old can get at least ¥750 per month as Supplemental Security Income (SSI) [45]. Considering this generation of elders had experienced full employment period of China, it is likely that most of them are entitled to employment pension, which would be much more than the SSI. Therefore, money from children matters less. It also bears mentioning that, in Chinese cultural tradition, children are obligated to give their parents money and gifts as a way of showing respect. Therefore, money from family members alone may not make a difference in an elder’s SRH.

### Neighbourhood economic status

Neighbourhood economic status appears to be an important determinant of SRH. Respondents in wealthier neighbourhoods tend to have better SRH, while those in poorer neighbourhoods tend to have poorer SRH. Respondents from neighbourhoods with high economic status tend to meet with their adult children more often (72.3% meet with their children everyday vs. 56.5% and 58.6% in communities with medium and low economic status, respectively). They are also contacting with their neighbours more often (93.5% reported weekly contact vs. 84.6% and 87.9% in communities with medium and low economic status, respectively). Residents of wealthier communities attend more gatherings with their relatives and friends (32.7% do so weekly vs. 12.4% and 10.1% in communities with medium and low economic status, respectively). Meanwhile, only 4.4% of the respondents in wealthier communities responded that they have negative feelings about their current lives vs. 4.8% and 7.4% in communities with medium and low economic status, respectively. Finally, this group also has better SRH—only 9% of them rated “poor” as their health status vs. 15.4% and 17.4% from communities with medium and low economic status, respectively.

Undoubtedly respondents from wealthier neighbourhoods enjoy better living environment, which ensures the quality of life of the residents. A better living environment in turn enables them to host family, relatives and friends; and these interactions have positive association with their health and social life. Since elders living in wealthier communities tend to be better educated, they are likely to have had better income jobs and have better pension; thus, they tend to be more optimistic about their lives as well as their overall wellbeing, and these positive attitudes influenced their SRH outcomes. This finding is not surprising because health, and in particular SRH, is strongly associated with socio-economic conditions one is in. Similarly, prior studies have explored the link between neighborhood and health. They all found neighborhood is an important element of living condition, which may influence health in two ways: first, through health behaviors, attitudes and healthcare utilization, and influencing health status; second, through environmental quality and community resources. Thereby, future policymaking may focus on community environment for improve the elders’ heath status [46–48].

### Physical and mental health status

With regard to physical health, the overall prevalence of chronic disease among respondents was nearly 80%, which is 20% higher than the rate reported in the Fourth Household Health Survey in China. The five most commonly reported chronic diseases in this study were hypertension, heart disease, diabetes, cataracts and cerebrovascular disease. All of these diseases are a serious issue for public health in China. Chinese scholars have identified hypertension and coronary heart disease as main threats to the health of the elders [49].

In this study, only 1.9% respondents had poor ADLs, which is a much lower rate than that of the elders in other parts of China [50]. However, consistent with previous studies, both uninvariate and multivariate analyses show that respondents with lower ADLs scores tended to have lower SRH scores [51]. Chronic disease is a key factor leading ADLs dependence, and thus, chronic disease prevention and control are crucial for improving the elders’ health status [52].

Multiple regression analysis found limited influence of demographic and health risk behaviour variables on SRH. The most influential variables are mental and physical health. Respondents who reported feelings of loneliness tended to be widowed women over 80 years of age, with low educational attainment, and no outdoor activities. These respondents rarely meet with their children (less than once a year), never take part in community activities, feel nervous constantly, have lower ADLs scores, and tend to have low SRH scores.

This study’s findings are consistent with strong epidemiological and physiological evidences in the literature that social isolation is a particularly important risk factor affecting elders’ SRH. Higher levels of depression among elders is associated to poorer health-related quality of life [53], and loneliness have a significant impact on physical health [54, 55].

Despite the elders’ relatively poorer health condition, this study shows that most of elders residents in Shanghai are satisfied with their lives.

#### Limitations

This study should be interpreted in light of the following limitations. First, this cross-sectional study established correlations but not causal relations among social support, health condition and SRH. We anticipate future research in the area to further elucidate these relationships. Second, there was no meaningful gender analysis conducted due to the gender imbalance in survey respondents.

## Conclusions

This is the first study on the SRH of Shanghai elders and its determinants such as socio-demographics, lifestyle, neighbourhood economic condition and health status. The main findings of this study include: nearly two-fifths of the respondents reported their own health as good; respondents from wealthier neighbourhoods tend to enjoy more social support, are more optimistic about their lives, and their SRH scores tend to be higher; respondents who receive less social support and have limited contacts with their children tend to feel lonely, and tend to have lower SRH scores; respondents with low ADLs scores tend to also have low SRH scores, as well as those with mental health concerns. Unlike in previous studies, financial support from family members did not translate into higher SRH score in Shanghai, which we tribute to Shanghai’s social security system and the Chinese cultural tradition that support parents is children’s obligation.

These findings illustrate the main determinants of SRH, and highlight the important elements that keeping elders healthy—both physically and mentally. This study sheds light on the general condition of the older population of Shanghai as reflected by their SRH. Our findings will also be valuable to policymakers in planning and allocating appropriate resources for the wellbeing of Shanghai’s ever-growing older population, and they will also help other scholars to further explore relevant social issues and help practitioners develop effective intervention strategies. Further studies adding to these findings are anticipated.

## Abbreviations

ADLs: activities of daily living
SRH: Self-rated Health

## References

[1] Gao Li-Ping. The Health Condition and lts Determinants of The Elderly Population in Shandong Province. Ph.D Thesis. Shandong University. Shandong. 2011.

[2] World bank database. 2016. http://data.worldbank.org/indicator/SP.POP.DPND.OL?view=chart. Accessed 2016.

[3] United Nations (2016). Let the young and old happy with the joy of the goal of aging.

[4] Organization W H. China country assessment report on ageing and health [J]. Working Papers, 2016.

[5] The national bureau of statistics of China. Main data bulletin of the Sixth national census. China News. 2011. http://www.chinanews.com/gn/2011/04-28/3004638.shtml. Accessed 28 Mar 2011.

[6] Chen Xian hua. Study on assessment of multi-health functional status and analysis of their impact factors of the community-dwelling elderly. Ph.D Thesis. Huazhong University of science and Technology. Hubei. 2009.

[7] Economic Development Analysis Report of Shanghai Municipal People's Government Development Research Center [J]. Science Development. 2017;1:5–14.

[8] Committee on Aging Affairs of Shanghai: Information of the eldely people in Shanghai. 2010.http://www.shanghai60.org.cn. Accessed 18 Mar 2011. http://www.shanghai.gov.cn/nw2/nw2314/nw2315/nw4411/u21aw1118419.html.

[9] Tan Feng-Yong. Effect of daily activities on successful aging in older adults. Ph.D Thesis. East China Normal University. Shanghai. 2011.

[10] The proportion of aging population in Shanghai will be higher than that of Japan [J]. International Finance. 2016;8:77–7.

[11] Chen Y, While AE, Hicks A (2015). Self-rated health and associated factors among older people living alone in shanghai[J]. Geriatr Gerontol Int.

[12] World Health Organization. Health interview surveys: Towards international harmonization of methods and instruments. Copenhagen: WHO Regional Office for Europe. 1996. 8857196

[13] Trachte F, Geyer S, Sperlich S. Impact of physical activity on self-rated health in older people: do the effects vary by socioeconomic status?[J]. J Public Health. 2016;38(4):754-59. 10.1093/pubmed/fdv198 28158738

[14] Kuosmanen K, Rovio S, Kivipelto M, Tuomilehto J, Nissinen A (2016). Determinants of self-rated health and self-rated physical fitness in middle and old age[J]. European Journal of Mental Health.

[15] Dai Y, Zhang CY, Zhang BQ (2016). Social support and the self-rated health of older people:a comparative study in Tainan Taiwan and Fuzhou Fujian province[J]. Medicine.

[16] Meng X, D’Arcy C (2016). Determinants of self-rated health among Canadian seniors over time: a longitudinal population-based study[J]. Soc Indic Res.

[17] Medeiros SM, Silva LSR, Carneiro JA, et al. Fatores associados à autopercepção negativa da saúde entre idosos não institucionalizados de Montes Claros, Brasil [J]. Ciênc Saúde Coletiva. 2016;21:3377-86. ISSN 1413-8123. 10.1590/1413-812320152111.18752015 27828571

[18] Golini N, Egidi V. The latent dimensions of poor self-rated health: how chronic diseases, functional and emotional dimensions interact influencing self-rated health in Italian elderly[J]. Soc Indic Res. 2016;128(1):321–39.

[19] Jing H, Yi WQ, Yong LM (2009). Health status analysis of the elderly in China. Intelligence.

[20] Gerontology research group in Fujian province. Research report about "empty nesters" in urban and rural areas in Fujian province. 2009. http://www.cnca.org.cn/default/iroot1000610000/4028e47d208847f601208dcO9c970093.html. Accessed 1 Wed 2009.

[21] Shah S, Vanclay F, Cooper B (1989). Improving the sensitivity of the Barthel index for stroke rehabilitation. J Clin Epidemiol.

[22] Mossey JM, Shapiro E (1982). Self-rated health: a predictor of mortality among the elderly.[J]. Am J Public Health.

[23] Jylhä M (2009). What is self-rated health and why does it predict mortality? Towards a unified conceptual model. Soc Sci Med.

[24] A simple measure with complex determinants:investigation of the correlates of self-rated health in older men and women from three continents. 10.1186/1471-2458-12-649 PMC3490893 22888996

[25] Bardage C, Pluijm SMF, Pedersen NL (2005). Self-rated health among older adults: a cross-national comparison. European Journal of Ageing.

[26] Kunst AE, Bos V, Lahelma E (2005). Trends in socioeconomic inequalities in self-assessed health in 10 European countries. Int J Epidemiol.

[27] Barros MBA, Zanchetta LM, Moura EC (2009). Self-rated health and associated factors, Brazil, 2006. Rev Saude Publica.

[28] Verropoulou G (2012). Determinants of change in self-rated health among older adults in Europe: a longitudinal perspective based on SHARE data. European Journal of Ageing..

[29] Nummela O, Sulander T, Rahkonen O (2008). Associations of self-rated health with different forms of leisure activities among ageing people. International journal of public health.

[30] Huang Xiao-Xia, Yan Yan the effect of family support on SRH of community-dwelling old people in Changsha. Chinese Journal of Gerontology. 2009;29(23):3090–3092 for the influence of self-evaluation of health in changsha city community old people.

[31] Jian xin L (2007). Study on the relationship between the quality of life and social support of elderly people. Population Research.

[32] QingQing M, Hong ZT (2010). Analysis of the influence factors of the elderly health self-assessment. Journal of Peking University(Health Sciences).

[33] Zhang XJ, Sun YH, Ni JF (2006). Analysis on dependency of daily life and it's relationship with chronic disease among rural elderly population in Anhui Province. Zhonghua liu xing bing xue za zhi= Zhonghua liuxingbingxue zazhi.

[34] Vuorisalmi M, Pietilä I, Pohjolainen P (2008). Comparison of self-rated health in older people of St. Petersburg, Russia, and Tampere, Finland: how sensitive is SRH to cross-cultural factors?. European Journal of Ageing..

[35] Girón P (2012). Is age associated with self-rated health among older people in Spain?. Cent Eur J Public Health.

[36] Wei HU-H, Jiao LI-Y (2011). Study on the health status of senior citizens in China and its influencing factors-based on the ordered probit model. Journals of Shanxi Finance and Economics university.

[37] Liu H, Chao JQ, Yang YC (2009). Determinants of self-rated health among the elderly and their extents. Chin Gen Pract.

[38] Dong W (2008). Cost containment and access to care: the shanghai health care financing model. The Singapore Economic Review.

[39] Lin G, Chun QX (2006). Analysis on influence factors of self-rated health of the elderly in our country. Population Journal.

[40] Maddox G L, Douglass E B. Self-assessment of health: a longitudinal study of elderly subjects. Journal of health and social behavior. 1973:87–93. 4708417

[41] Holmes WR, Joseph J (2011). Social participation and healthy ageing: a neglected, significant protective factor for chronic non communicable conditions. Glob Health.

[42] Lu S, Shu-Zhuo L (2006). The effect of intergenerational support on rural elderly people’s SRH. Chin J Gerontol.

[43] Sugiyama T, Thompson CW (2007). Older people's health, outdoor activity and supportiveness of neighbourhood environments. Landsc Urban Plan.

[44] Xiao-Xia H, Yan Y (2009). The effect of family support on SRH of community-dwelling old people in Changsha. Chin J Gerontol.

[45] Bo Feng. 2016 Pensions raised about 6.5% [J]. Old comrades monthly, 2016 (5): 14–14.

[46] Ellen IG, Mijanovich T, Dillman KN (2001). Neighborhood effects on health: exploring the links and assessing the evidence[J]. Journal of Urban Affairs.

[47] Braveman P, Cubbin C, Egerter S (2011). Neighborhoods and health[J].

[48] Americans M (2008). Where we live matters for our health : the links between housing and health[J].

[49] Hui Rong ,Zhang Hua Li, Zhang Ru Ying, LiuYan Jun. Xi 'an. Investigation of the population distribution characteristics of 1811 cases of elderly with chronic disease. Chinese Journal of Nursing 2003;38(2):153–155.

[50] Lv ZHOU (2012). Research on relationship between elders’ socioeconomic status and their activities of daily living failure. Population and Development.

[51] Menec VH, Shooshtari S, Lambert P (2007). Ethnic differences in self-rated health among older adults a cross-sectional and longitudinal analysis. Journal of Aging and Health.

[52] Reed RL, Battersby M, Osborne RH (2011). Protocol for a randomised controlled trial of chronic disease self-management support for older Australians with multiple chronic diseases. Contemporary clinical trials.

[53] Chan SW, Shoumei JIA, Thompson DR (2006). A cross-sectional study on the health related quality of life of depressed Chinese older people in shanghai. International journal of geriatric psychiatry..

[54] Luanaigh CÓ, Lawlor BA (2008). Loneliness and the health of older people. International journal of geriatric psychiatry.

[55] Lacruz ME, Emeny RT, Haefner S (2012). Relation between depressed mood, somatic comorbidities and health service utilisation in older adults: results from the KORA-age study. Age Ageing.

